# Transparency induced in opals via nanometer thick conformal coating

**DOI:** 10.1038/s41598-019-47963-2

**Published:** 2019-08-06

**Authors:** Guoliang Shang, Kaline Pagnan Furlan, Robert Zierold, Robert H. Blick, Rolf Janßen, Alexander Petrov, Manfred Eich

**Affiliations:** 1 Institute of Optical and Electronic Materials, Hamburg University of Technology, Eissendorfer Strasse 38, 21073 Hamburg, Germany; 2 Institute of Advanced Ceramics, Hamburg University of Technology, Denickestrasse 15, 21073 Hamburg, Germany; 3 Center for Hybrid Nanostructures, Universität Hamburg, Luruper Chaussee 149, 22607 Hamburg, Germany; 4 ITMO University, 49 Kronverkskii Ave., 197101 St. Petersburg, Russia; 5 Institute of Materials Research, Helmholtz-Zentrum Geesthacht, Max-Planck-Strasse 1, Geesthacht, D-21502 Germany

**Keywords:** Photonic crystals, Optical materials and structures

## Abstract

Self-assembled periodic structures out of monodisperse spherical particles, so-called opals, are a versatile approach to obtain 3D photonic crystals. We show that a thin conformal coating of only several nanometers can completely alter the reflection properties of such an opal. Specifically, a coating with a refractive index larger than that of the spherical particles can eliminate the first photonic band gap of opals. To explain this non-intuitive effect, where a nm-scaled coating results in a drastic change of optical properties at wavelengths a hundred times bigger, we split the permittivity distribution of the opal into a lattice function convoluted with that of core-shell particles as a motif. In reciprocal space, the Bragg peaks that define the first Brillouin zone can be eliminated if the motif function, which is multiplied, assumes zero at the Bragg peak positions. Therefore, we designed a non-monotonic refractive index distribution from the center of the particle through the shell into the background and adjusted the coating thickness. The theory is supported by simulations and experiments that a nanometer thin TiO_2_ coating via atomic layer deposition (ALD) on synthetic opals made from polystyrene particles induces nearly full transparency at a wavelength range where the uncoated opal strongly reflects. This effect paves the way for sensing applications such as monitoring the thicknesses growth in ALD *in-situ* and in real time as well as measuring a refractive index change without spectral interrogation.

## Introduction

Photonic crystals (PhCs) are periodic dielectric structures^1–6^. The periodic modulation of the dielectric leads to the appearance of a photonic band gap (PBG), which prohibits the propagation of light within certain wavelength region in certain or all directions. A PBG therefore allows to filter, confine and manipulate light^7,8^.

Among various fabricating procedures, synthetic opals produced by self-assembly are a versatile approach to obtain 3D PhCs. Here, monodispersed spherical particles are self-assembled into a close-packed face centered cubic (FCC) structure which has the {111} planes parallel to the substrate. The FCC geometry is represented by a body centered cubic (BCC) lattice of Bragg peaks in reciprocal space with closest Bragg peaks defined by {111} planes. The so-called Bragg condition should be fulfilled to efficiently reflect light from crystalline planes. This condition includes the wavelength of light in the material, angle of incidence and interplane distance^3,4^ and can be visualized in reciprocal space by Ewald sphere construction^9,10^. The shift of the PBG with the change of the effective refractive index can be used for sensing applications^8,11–15^. Here, we provide an alternative approach to utilize the opal structure. Namely, we investigate not the shift of the PBG with the refractive index change, but the change of the PBG reflectivity strength. We show that the optical properties of the opal structure is very sensitive to the presence of a thin conformal coating on its surface, especially if the refractive index of this coating is larger than that of the particles. The coating can even lead to the elimination of the first PBG in all directions. Such a structure has several interesting applications. First, the transparent opal can keep its porosity in the wavelength range, thus providing a non-scattering porous medium. Second, the transparent opal is very sensitive to the refractive index change of the background/environment. In this case the PBG is not just shifted by the refractive index change, but switched on and off. Thus, this effect allows sensing without the need for spectrally selective sources, but by probing broadband transmission or reflection from the opal.

In the previous publications, the PBG strength variation with different conformal coatings was reported, but not explained theoretically^16–19^. In this work, we describe the effect using the first-order approximation^9,20^, which we also have recently applied to a photonic glass (PhG) structure^10^. In this approximation, the scattering properties of the structure are mainly defined by its Fourier transform (FT). Similar to what was done for the PhG structure, we split the opal structure into a lattice and a motif. The lattice defines the Bragg peak positions in the reciprocal space, which are weighted by the motif function. We show the possibility to eliminate the lattice peaks in reciprocal space by engineering the motif structure. When the first-zero position of the motif FT falls on the first peak position of the lattice FT, this peak will be eliminated and the bandgap will vanish. The consequence of this is that the opal changes its optical property from reflecting to transmitting in the specified wavelength range. Numerical simulations and experimental results confirm the theory.

## Results and Discussion

The light scattering properties of structures with permittivity perturbation ${\rm{\Delta }}\varepsilon (\overrightarrow{r})$ with respect to the background level can be predicted using the first-order approximation^20^. In our previous works, by using the Ewald sphere construction we have shown that the scattered power *P* from a scattering volume increases proportionally to the square of the absolute value of FT of ${\rm{\Delta }}\varepsilon (\overrightarrow{r})$ integrated over the Ewald sphere surface (ESS)^9,10,21^:

1 $$P={I}_{0}\frac{{\omega }^{4}}{16{\pi }^{2}{c}^{4}}\mathop{\int }\limits_{ESS}\,\frac{{| {\mathcal F} \{{\rm{\Delta }}\varepsilon (\overrightarrow{r})\}(\overrightarrow{k})|}^{2}}{{k}_{s}^{2}}g(\theta ){d}^{2}k$$

where *I*_0_ is the intensity of the incident plane wave of light, *θ* is the angle between scattered ${\overrightarrow{k}}_{s}$ and input ${\overrightarrow{k}}_{in}$ wavevectors and for unpolarized light $g(\theta )=(1+{\cos }^{2}\theta )/2$. The length of ${\overrightarrow{k}}_{in}$ is defined as *n*_*eff*_*ω*/*c*, where *ω* is the frequency and *c* is the speed of light in vacuum and *n*_*eff*_ the effective refractive index of the opal. The Ewald sphere is shifted from the origin of reciprocal space by $-\,{\overrightarrow{k}}_{in}$ and has a radius *k*_*s*_. Thus, the amplitude of ${\mathcal F} \{{\rm{\Delta }}\varepsilon (\overrightarrow{r})\}(\overrightarrow{k})$ should be small in a sphere with radius 2*k*_*s*_ to avoid scattering.

**Figure 1:**
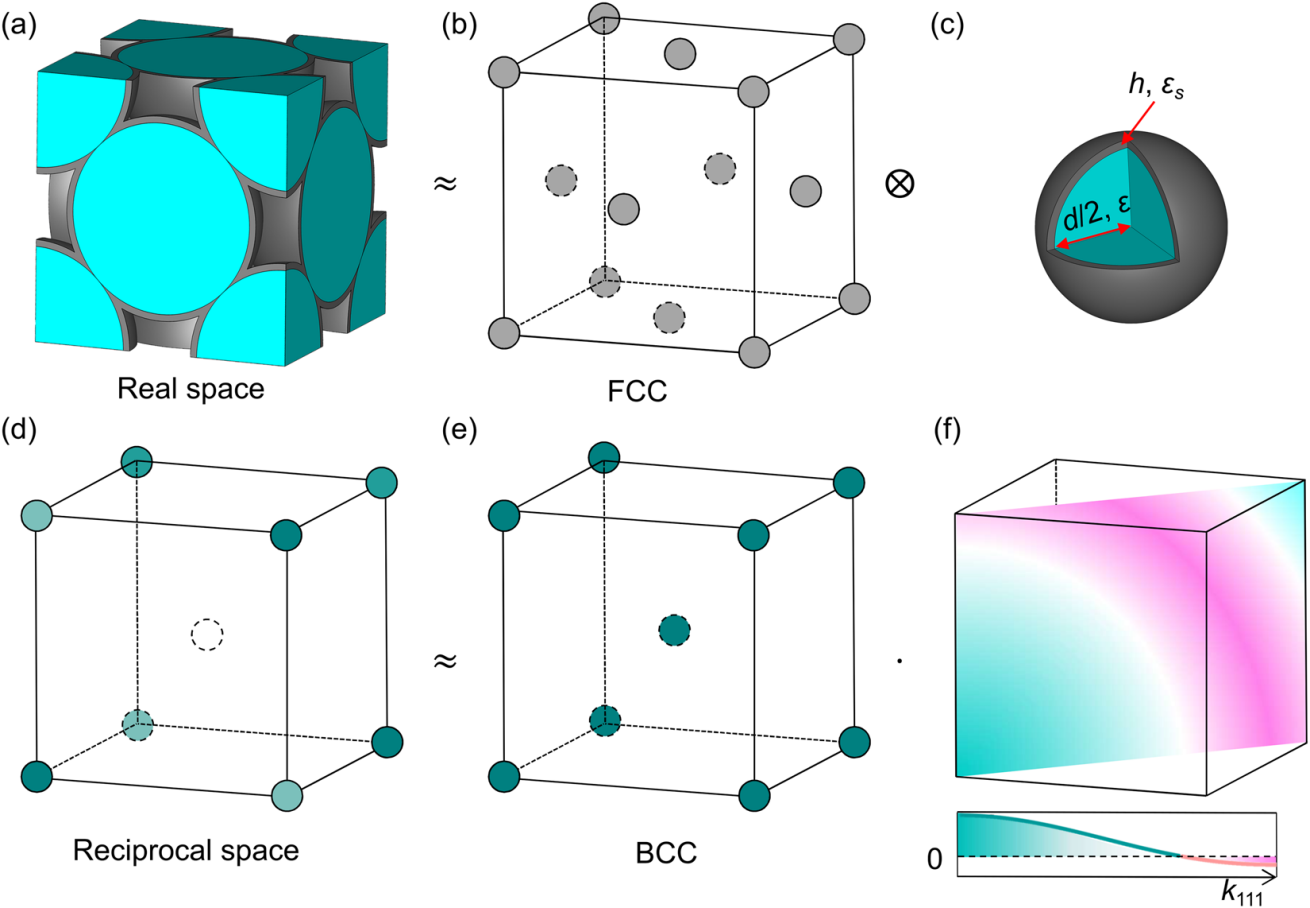
A conformally coated opal is schematically illustrated in real and reciprocal space. In real space the structure (**a**) can be seen as the convolution of the FCC lattice (**b**) with the motif (**c**). The motif can be simplified to a core-shell sphere by neglecting the contact points between spheres. Accordingly, the total FT of the structure (**d**) can be written as multiplication of the lattice FT (**e**) and the motif FT (**f**) where the amplitude distribution on the {110} plane is further shown.

The opal structure (Fig. 1a) can be seen as the convolution of the FCC lattice function $l(\overrightarrow{r})$ (Fig. 1b) with the motif function $m(\overrightarrow{r})$ of spherical particles (Fig. 1c). In reciprocal space, the FT of ${\rm{\Delta }}\varepsilon (\overrightarrow{r})$ for the whole structure (Fig. 1d) is the multiplication of the lattice FT ${ {\mathcal F} }_{l}(\overrightarrow{k})$ (Fig. 1e) and the motif FT ${ {\mathcal F} }_{m}(\overrightarrow{k})$ (Fig. 1f) ${\mathcal F} \,\{{\rm{\Delta }}\varepsilon (\overrightarrow{r})\}={ {\mathcal F} }_{l}(\overrightarrow{k})\cdot { {\mathcal F} }_{m}(\overrightarrow{k})$^10^. The FCC lattice function of the opal has a BCC lattice in reciprocal space (Fig. 1e). The closest 8 Bragg peaks have distance to the origin of 1.22 × 2π/*d*, where *d* is the sphere diameter. The motif function of a solid sphere with volume *V* and permittivity contrast Δ*ε* has the following radial function in reciprocal space^22,23^:

2 $${ {\mathcal F} }_{m}(k)={\rm{\Delta }}\varepsilon \frac{3[\sin (k\frac{d}{2})-(k\frac{d}{2})\cos (k\frac{d}{2})]}{{(k\frac{d}{2})}^{3}}V$$

This function is zero on a spherical surface with a radius of 1.43 × 2π/*d*. Thus the motif of the original opal based on solid spheres can never eliminate the first Bragg peaks in the reciprocal space. We now show that a conformal coating on the surface of the opal can change the motif such that its zero surface eliminates all first Bragg peaks of the BCC reciprocal lattice.

Figure 1 schematically illustrates the conformally coated opal structure in real and reciprocal space. The experimental opals (Fig. 1a) have a FCC lattice (Fig. 1b)^3,4^. At the same time, since the coating thickness is much smaller than the sphere size, we simplify the motif of the conformal deposited opal to a core-shell sphere by neglecting the contact points of the spheres. This simplification leads to an overestimation of the coating volume (see Supplementary Materials). The core-shell sphere has a core diameter *d*, employing a permittivity *ε* and a shell thickness *h* with a permittivity of *ε*_*s*_. Correspondingly, the opal structure has a BCC lattice (Fig. 1e) in the reciprocal space^4^. The closest Bragg peaks in opals are defined by {111} planes with a lattice constant of ${d}_{111}=\sqrt{\frac{2}{3}}d$.

**Figure 2:**
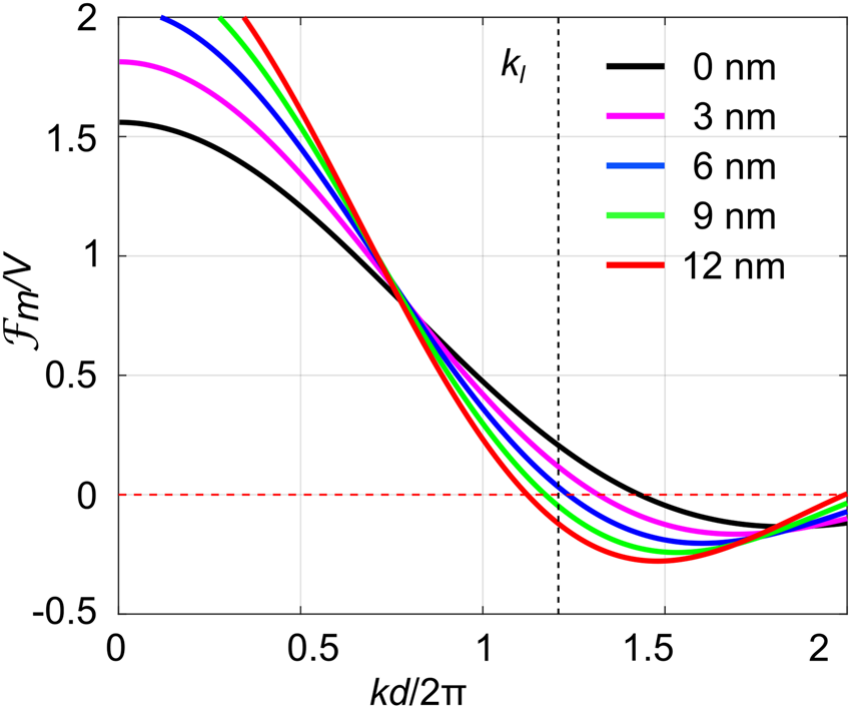
The normalized amplitude function ${ {\mathcal F} }_{m}/V$ of the FT of a spherical polystyrene core-shell particle of 172 nm diameter uncoated (black) and with a titania shell of 3 nm (purple), 6 nm (blue), 9 nm (green) and 12 nm (red) thickness adding to the radius. The background material is air. The horizontal axis corresponding to the radial wave number is normalize*d* by 2π/*d*. The vertical black dash line indicates the Bragg peak position.

Figure 2 shows the normalized amplitude $\frac{{ {\mathcal F} }_{m}}{V}$ of the FT of a core-shell sphere with parameters *d* = 172 nm, *ε* = 2.56 (*n* = 1.6), *ε*_*s*_ = 5.29 (*n*_*s*_ = 2.3) and the shell thickness *h* varying from 0 nm (uncoated) to 12 nm, respectively. As coating material we assume amorphous TiO_2_ with a refractive index of 2.3 and disregard the refractive index dispersion. This refractive index of the TiO_2_ coating produced by ALD was measured by ellipsometry on a flat reference for a wavelength of 632 nm. The horizontal axis corresponding to the radial wave number is normalized by 2π/*d*. The vertical black dashed line indicates the Bragg peak position. As can be seen from Fig. 2, the first-zero position of motif FT can be gradually moved to lower *k* region with respect to *k*_*l*_ by increasing the shell thickness *h*. This shift becomes possible only if the coating permittivity is larger or smaller than both, the permittivity of the sphere and of the background as we have shown in our previous publication^10^. The corresponding amplitudes at position *k*_*l*_ are 0.19, 0.11, 0.02, −0.05 and −0.12. The square of the amplitude will define the scattering strength of the corresponding Bragg peak. Thus the scattering strength can be significantly suppressed for a shell thickness of only 6 nm. This suppression is acting on all 8 closest Bragg peaks due to spherical symmetry of the motif function.

**Figure 3:**
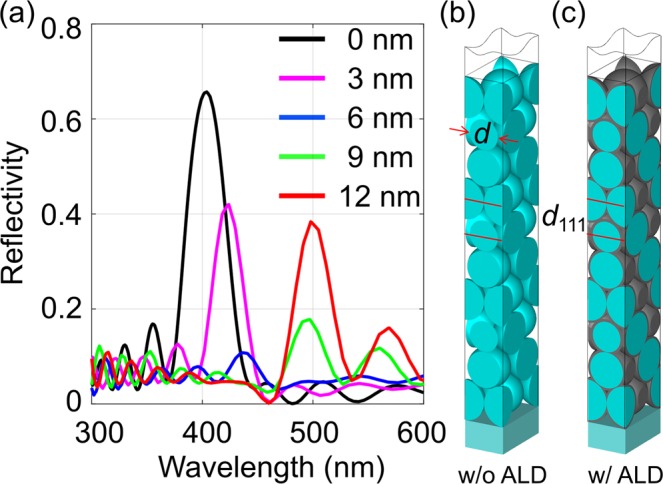
(**a**) Simulated reflectance spectra of the opal structure along [111] direction for uncoated (black) and different coating thicknesses of 3 nm (purple), 6 nm (blue), 9 nm (green) and 12 nm (red). The simulated structure of the opal without (**b**) and with (**c**) conformal coating. Periodic boundary conditions are used at vertical boundaries of the simulation volume. The opal has 10 {111} planes.

To confirm the theoretical predictions, we have numerically simulated the opals with different thicknesses of conformal titania coatings (without the core-shell simplification), as well as the reference opal (uncoated). Figure 3 shows the reflectance spectra of the brute-force 3D finite integration simulation of opals. The opal layers are lying on a glass substrate *n* = 1.5 and have air as background. The simulated opal structures have 10 {111} layers. The reflection peak *λp* shifts gradually to longer wavelength due to increase of the effective refractive index with increasing shell thickness^17^. The simulated transmittance spectra can be found in Fig. S2. It reveals that a minimal reflection will be observed somewhere between 6 and 9 nm coatings. In fact, slightly larger coating thicknesses are required in the simulation as compared to first order approximation with core shell particles, because the latter neglects the sphere contact areas and, thus, overestimates the coating volume. One of the most remarkable features of this effect is that the PBG disappears and then reappears again, which confirms the theoretical prediction. The band diagram (Fig. S4) of the uncoated and coated opal also verified this phenomenon that the band gap disappear and reappear with coating thickness at ΓL direction ([111] direction). Similarly, the band gap at ΓX direction will increase which also can be explained by the variation of the first zero point of the motif FT (Fig. S3d).

**Figure 4:**
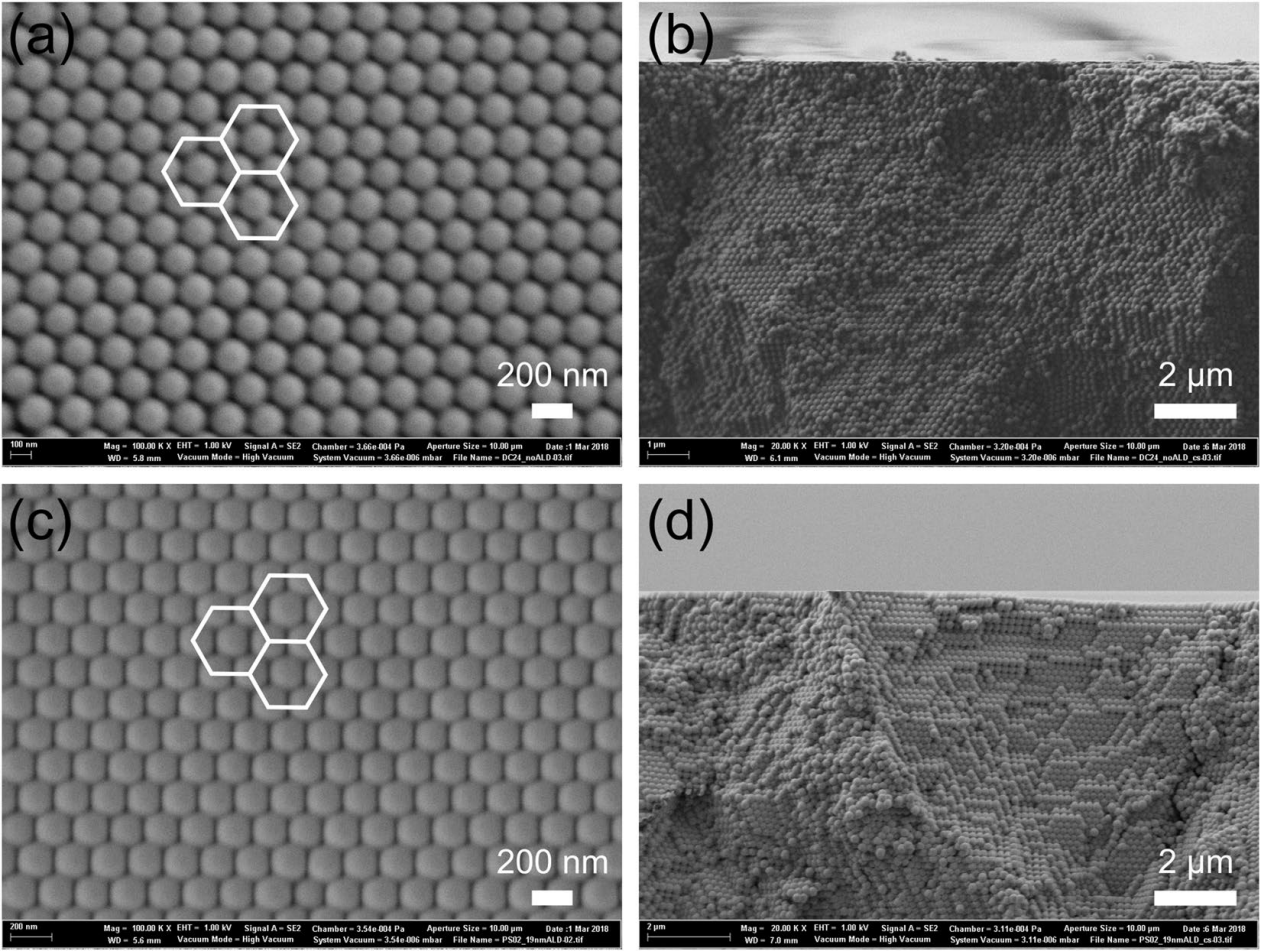
The structures of the prepared opal without (**a**,**b**) and with (**c**,**d**) conformal coating of TiO_2_. Top view (**a**) and cross-sectional (**b**) SEM images of the opal without conformal coating. Top view (**c**) and cross-sectional (**d**) SEM images of the opal with 19.1 nm (full infiltration) TiO_2_ conformal coating. The white hexagons in image (**a**,**c**) illustrate the close-packing and the {111} planes.

To experimentally verify the theory and the supporting simulations, we also performed conformal deposition of TiO_2_ on polystyrene (PS) opal by ALD. The PS spheres with diameter of 172 ± 6 nm were drop casted on a glass substrate. During the drying process, the PS spheres are self-assembling into an opal structure. The top view SEM image (Fig. 4a) shows hexagons inside the densely packed crystal of spheres which indicates the {111} planes, one of which is parallel to the substrate. Figure 4b shows the cross-sectional SEM image of the opal without conformal coating. The surface (Fig. 4c) and the cross-sectional (Fig. 4d) SEM images for 19.1 nm TiO_2_ conformal coating show that the interstitials between the spheres are decreased.

**Figure 5:**
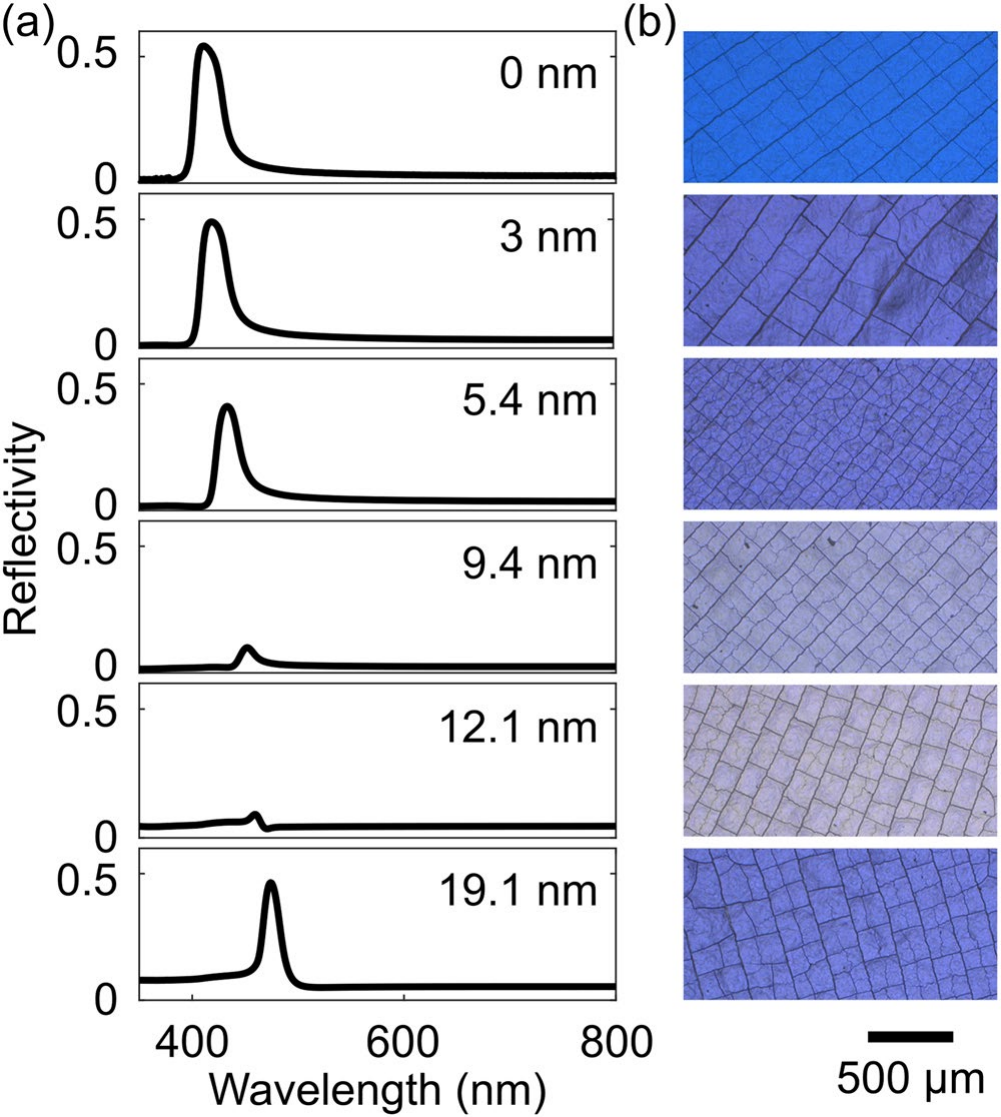
(**a**) The measured specular reflectance spectra of the opal films with a total height of ~15 µm conformally coated with different thicknesses of TiO_2_. (**b**) The corresponding microscope images. The rectangular pattern on the film surfaces stem from macroscopic drying cracks originated from the self-assembly process.

Figure 5 shows the measured specular reflectance spectra and the corresponding color appearance for uncoated and coated samples with TiO_2_ thickness of 3 nm, 5.4 nm, 9.4 nm, 12.1 nm and 19.1 nm, respectively. The complementary transmittance spectra are shown in the supplementary information (Fig. S3). The peak reflectivity (Fig. 5a) gradually decreases when the deposition thickness increases from 0 nm to 9.4 nm. At about 12.1 nm coating thickness, the PBG has nearly disappeared, then reappears again for thicker coatings. For 19.1 nm coating thickness we clearly see reappearance of the peak. These experimental results confirm the theoretical prediction of the PBG shift, extinction and reappearance mechanism. In addition, the experimental reflection spectra in Fig. 5 show a trend that the sharper reflection transient as well as the low reflectivity interchanges from short wavelength region to the longer wavelength region with increasing thickness. This phenomenon is consistence with the theoretical prediction from first order approximation. The zero point shifts from the long *k* (short wavelength) to short *k* (long wavelength) region across the Bragg peak (Fig. 2), that lead to a sharp transition in the spectrum first at the short wavelength edge then in the long wavelength edge. The low reflection region also changes accordingly. The same effect was previously utilized by us to obtain highly saturated structural color in PhG^10,24^. However, the simulation reflection spectra (Fig. 3a) are slightly different from experiment (Fig. 5a). The simulations show Fabry-Perot resonances in the opal film which complicate the spectra. In the experiments the Fabry-Perot oscillations are averaged out due to the thickness variations of the opal film within the measurement spot.

The small shifted position of the suppressed PBG between experimental value and simulation might be attributed to several experimentally related reasons and remains open for further investigations: First, it is reported that the growth mechanisms of ALD films onto polymeric surfaces may vary from inorganic silicon wafers due to the different surface species^25^. Second, an emphasized intrinsic growth inhibition due to a lack of water excess inside the opal will reduce the growth rate^26^. In addition, the possible limited diffusion time of the precursor gas into the porous network would also lead to a thickness gradient from top to bottom. All three facts may contribute to a reduced growth inside the opal. Thus leading to a smaller shell thickness compared to the thickness measured on a plain Si reference^16^.

Due to the reflection caused by the PBG, the sample shows a very bright blue color (Fig. 5b) when there is no coating at all. The blue color impression becomes very pale for the 12.1 nm TiO_2_ coating. The whitish impression of the reflected light is due to the residual broadband scattering originating from the imperfect structure, known defects, such as cracks, dislocations and vacancies in the opaline 3D structure^27^. When we further increase the coating thickness, the PBG effect is restored. With this, an effective narrow band reflection takes place again and the sample color reappears as bright blue with a different blue tone which is caused by a slight wavelength shift of the PBG. The experimental studies confirm the theoretical prediction and simulation results. In fact, we showed that the strength of the PBG in opals can be controlled or even completely suppressed by nanometer thin conformal coatings. The presented effect can be used in future to directly target *in-situ* ALD coating thickness monitoring (see supplementary material, Fig. S5) in opaline nanostructures to shed light onto the growth behavior, diffusion processes, and precursor interaction with the substrate. Also, the coating can be used to make the opal transparent for a background medium of choice. For example the opal transparent with water background can be used for sensing. In this case small changes in fluid refractive index will result in large relative change of reflection. The sensing in this case does not require a spectrometer as the position of the reflection peak does not have to be tracked (see supplementary material, Fig. S6).

## Conclusion

In conclusion, we theoretically and experimentally described the effect of transparency in synthetic opal structures induced by thin conformal coatings. Our approach is based on precise design of the reciprocal space properties of the motif. Namely, the repeating motif of the opal, a coated sphere in our case, can be used to eliminate the Bragg peaks of the lattice in reciprocal space. The refractive index of the coating has to be larger or smaller than the refractive index of both, the sphere and the background medium. Here, we show experimentally that a polystyrene synthetic opal can be made transparent with approximately 10 nm thick conformal titania coating deposited via ALD. The strong sensitivity of the PBG strength in opals to such a thin conformal coating can be used for sensing applications, such as *in-situ* ALD coating measurement or as a refractive index sensor without spectral interrogation.

## Materials and Methods

### Simulations

Under normal incidence, the light reflectance (*R*) and transmittance (*T*) of the opal layer were simulated by using the finite integration technique (FIT),which is integration-based, implemented in CST Microwave Studio^28^. The opals have 10 layers of the close-packed {111} planes. The refractive index of the PS is assumed as *n* = 1.6. The refractive index of TiO_2_ coating is *n*_*s*_ = 2.3, which comes from ellipsometric measurements of ALD films deposited on planar reference substrates. The opals are then excited by a broad band plane wave vertically impinging from air. The lateral sides of the simulation volume are periodic boundary conditions. The opal layer is lying on the substrate glass material with refractive index equal to *n* = 1.5 to reproduce the experimental conditions. The homogeneous substrate material is then terminated by an open boundary condition. The incident light can exit the simulation volume only through the open boundaries at the top and the bottom, and monitored by a dense grid of field probes. The field probes collect the time-dependent signal which can then be split into the incident signal and the reflected signal. Then, the reflected and transmitted powers are calculated as Poynting vector of the steady state integration over the upper and lower boundaries, respectively.

### Sample preparation

Prior sample preparation the soda lime substrates were placed in a Mucasol 1% solution (Sigma Aldrich) and cleaned in an ultrasonic bath for 1 h, followed by brushing, rinsing with ultrapure water (MiliQ) and drying under nitrogen flow. Direct opals were produced by drop casting of 100 µL of polystyrene spheres water-based suspension onto the previously cleaned and heated (60 °C) soda lime glasses (Thermo Fischer Scientific), followed by drying at the same temperature for 15 minutes. The polymer particle size and suspension concentration were 172 ± 6 nm (Microparticles GmbH) and 50 mg.mL^−1^, respectively. The direct opals were then infiltrated with titanium oxide by ALD process under a full exposure mode with constant flow of nitrogen (15 sccm) in a home-made reactor (Universität Hamburg, Physics Department, CHyN - Center for Hybrid Nanostructures) utilizing titanium Iso-propoxide (TTIP, Sigma Aldrich) heated up to 85 °C and deionized water at room temperature. The base pressure of the ALD system connected to an Edwards iQDP80 scroll pump was 9E-2 mbar. Pulse, diffusion/exposure and purge times were chosen based on past experiments to be 2/45/90 and 0.5/45/90 seconds for TTIP and H_2_O, respectively^29^. As a consequence, the duration of one cycle resulted to 272.5 s in total assuring a conformal infiltration of the opaline structure without cavities or chemical vapor deposition inside. An average growth of 0.6 Å per cycle —determined on a planar reference— was achieved and the total number of cycles was designed according to the desired film thickness, ranging from 3 to 19 nm.

### Measurements

The thickness and refractive index of the resulting coatings were determined by spectral ellipsometry (SENProTM, SENTECH Instruments GmbH) on a Si reference wafer placed close to the opals in the ALD cycle. For analyzing the ellipsometer data, a Cauchy model was used to fit the data for the titania layer leading to a mean refractive index of 2.29+/−0.03 at a wavelength of 632.8 nm being in good agreement with previous work^29^. Note, only samples with thicknesses >8 nm and additional reference samples run under identical conditions were taken into account. The specular reflectance and transmittance spectra between 300 nm and 800 nm are measured using UV/VIS spectrometer (Lambda 1050, Perkin Elmer) with URA and integrating sphere accessories. The digital images were taken by Zeiss microscope LSM 700 with Axiocam 105 color camera with color temperature of 3200 K illumination and 5× objective lens.

## Supplementary information

supplementary materials
