# Supplementary material for: Transparency induced in opals via nanometer thick conformal coating
Source: Sci Rep. 2019 Aug 6;9:11379. doi: 10.1038/s41598-019-47963-2 (PMC6684641; doi:10.1038/s41598-019-47963-2)
Supplement: Supplementary file 1 — supplementary materials [file 41598_2019_47963_MOESM1_ESM.pdf]

Transparency induced in opals via nanometer thick conformal coating

Guoliang Shang,[1,] Kaline Pagnan Furlan,[2,3] Robert Zierold,[3] Robert H. Blick,[3] Rolf Janßen,[2] Alexander Petrov,[1,4] and Manfred Eich[1,5]*

[1]Institute of Optical and Electronic Materials, Hamburg University of Technology, Eissendorfer Strasse 38, 21073 Hamburg, Germany

[2]Institute of Advanced Ceramics, Hamburg University of Technology, Denickestrasse 15, 21073 Hamburg, Germany

[3]Institute of Nanostructure and Solid State Physics and Center for Hybrid Nanostructures, University of Hamburg, Luruper Chaussee 149, 22607 Hamburg, Germany

[4]ITMO University, 49 Kronverkskii Ave., 197101, St. Petersburg, Russia

[5]Institute of Materials Research, Helmholtz-Zentrum Geesthacht, Max-Planck-Strasse 1, Geesthacht, D-21502, Germany

**guoliang.shang@tuhh.de*

Supplementary materials:

1. Contact points

In our consideration (Figure S1a) for the coating thickness *h*, the volume of the spherical cap which is lost at the contact point (shaded zone) is:

$$\Delta V = \frac{\pi h^2}{3}\left[\frac{3(d+2h)}{2} - h\right]$$

The shell volume *V* of a core-shell sphere is:

$$V = \frac{4\pi}{3}\left[\left(\frac{d}{2}+h\right)^3 - \left(\frac{d}{2}\right)^3\right]$$

The missed shell volume for a point contact and for 12 contacts (due to the FCC structure) compared to the core-shell sphere is shown in Fig. S1b. In the real structure the number of contact can decrease due to slight deviations in the size of the particles.

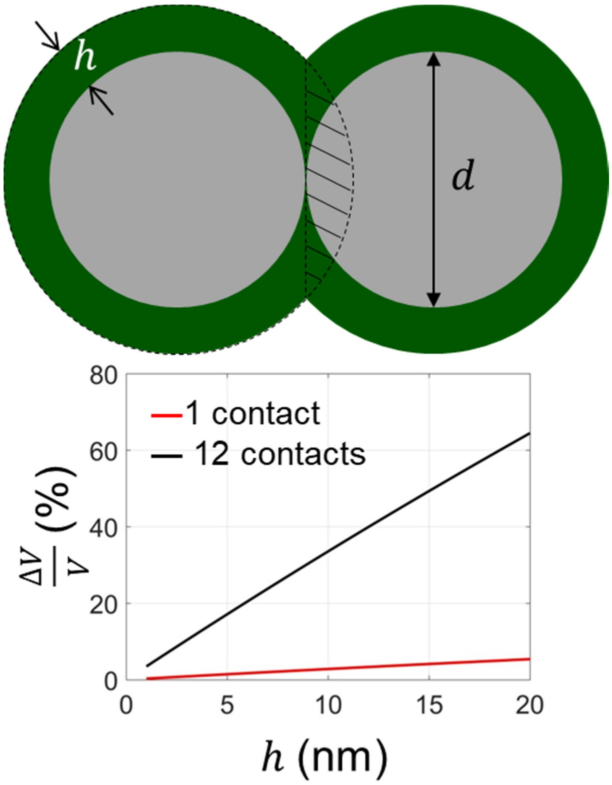

Figure S1. Overestimation of the volume for the motif simplified as a core-shell sphere. (**a**) The missed shell volume is the volume of the spherical cap with height of h. (**b**) The calculated $\frac{\Delta V}{V}$ in percent depending on the coating thickness h for sphere radius d=172 nm for 1 contact and 12 contacts.

2. Transmission simulation and experimental spectra

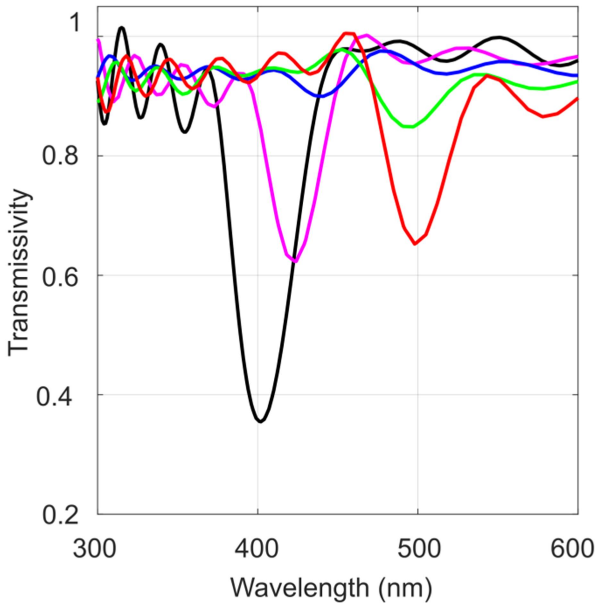

Figure S2. The simulated transmittance spectra of the opal with different coating thickness *h*. The PBG disappears when the coating thickness is 6 and 9 nm, and then reappears when the coating is 12 nm.

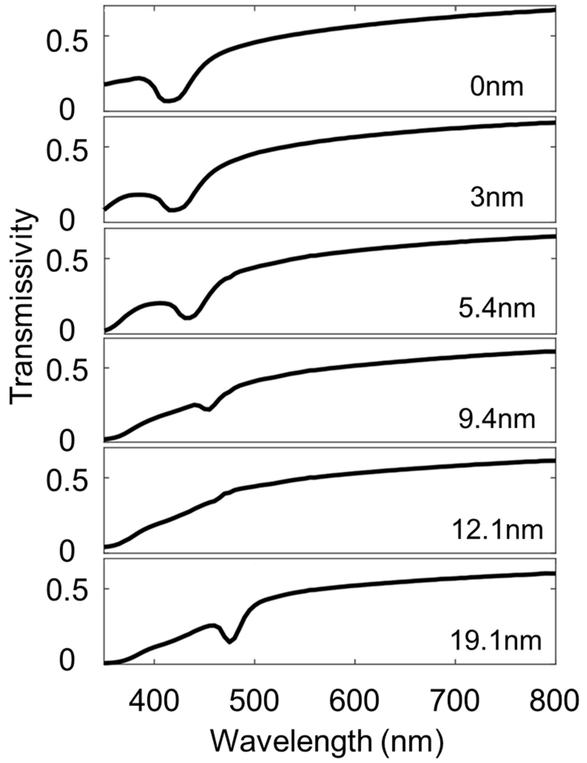

Figure S3. The measured total transmittance spectra of the opal structure with different thickness of TiO_2. The transmission drop at short wavelength is attributed to defects in the

self-assembled opal and the increasingly scattering from opal. The additional transmittance drop of conformal deposited samples compared to not deposited one at wavelengths below 360 nm is from the light absorption of TiO_2.

3. Band diagrams

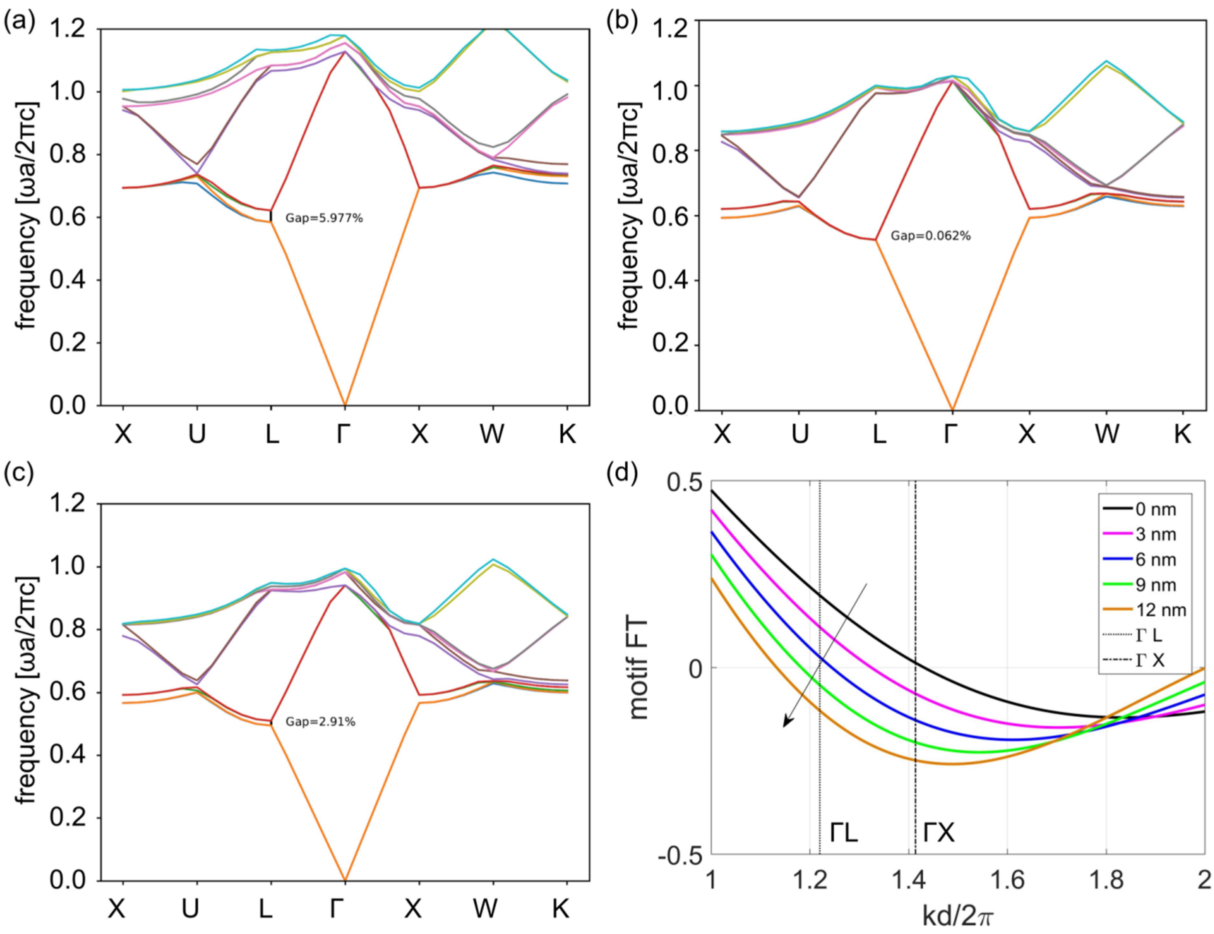

Figure S4. The band structure for the opal without (a), with a 7.5 nm (b) and 12 nm (c) conformal coating of TiO_2. (d) The normalized amplitude function $\mathcal{F}_m/V$ of the FT of a spherical polystyrene core-shell particle of 172 nm diameter uncoated (black) and with a titania shell of 3 nm (purple), 6 nm (blue), 9 nm (green) and 12 nm (red) thickness adding to

the radius. The background material is air. The vertical black dash line indicates the Bragg peak position at ΓL and ΓX direction.

The photonic band gap will shrink and disappear also in the band diagram. Band diagram calculation takes into account all effects, but the first order approximation presented here helps to explain why the band gap and thus the reflection peak disappear. The band structure (Figure S3) of direct opal and opal with 6 nm conformal coating is calculated by using the MPB software package from MIT (https://mpb.readthedocs.io/en/latest/). The direct opal without conformal coating (Figure S3a) shows a larger bandgap width (defined as band gap width divided by the central frequency, $\Delta\omega/\omega$) of 5.977% along the ΓL direction ([111] direction). When the structure is coated with a 7.5 nm TiO_2 film with a refractive index of 2.3 (Figure S3b), the band gap width $\Delta\omega/\omega$ decreases to 0.062%. The band gap then will increase to 2.910% when the coating thickness increase to 12 nm (Figure S3c). The band gap in the ΓL direction decreasing and then increasing with coating thickness is consistent with the first order prediction. Similarly, the band gap at ΓX direction is almost zero without coating and opens with coating deposition which also can be explained by the shift of the first zero point of the motif FT (Figure S3d).

4. Reflection peak position and amplitude

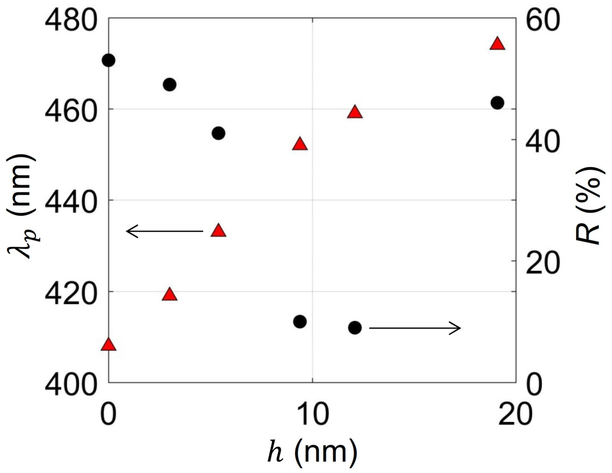

Figure S5. The peak positions (triangle) and reflectivity (circle) of the opal with different TiO_2 coating thickness. The corresponding peak positions are 408, 419, 433, 452, 459 and 474 nm, while the reflectivity are 53%, 49%, 41%, 10%, 9% and 46%. The maximal sensitivity to the coating thickness is achieved at approximately 7 nm and corresponds to 10% reflection change per nanometer.

5. Tuning of the transparency window to the refractive index of the background medium

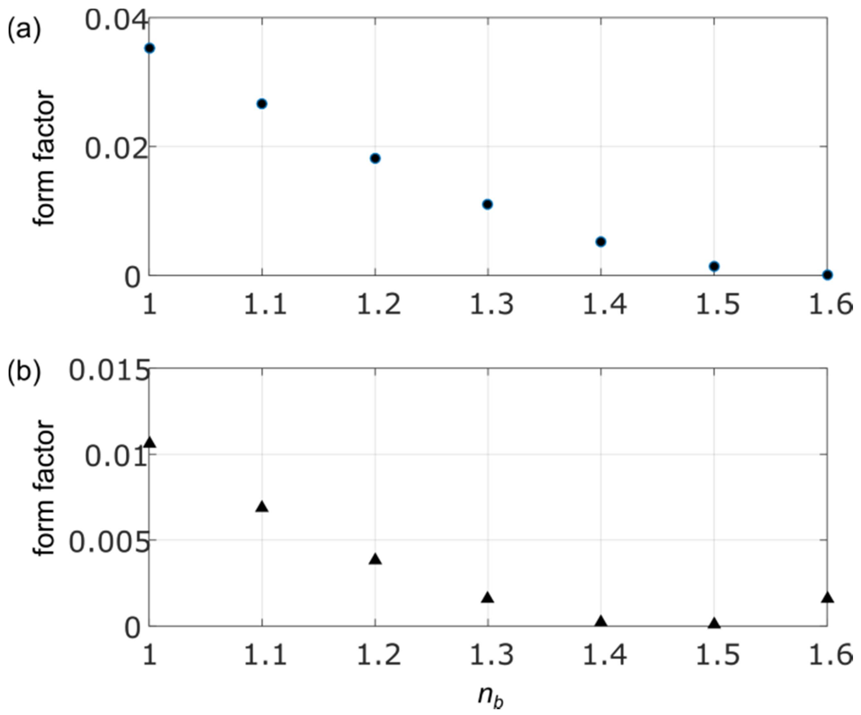

Figure S6. The form factor ($\mathcal{P} = \frac{\mathcal{F}_m{}^2}{V}$) at the Bragg peak as the function of the background refractive index (n_b) for the polystyrene opal with titania ALD coating of 0 nm (a), 3 nm (b).

Figure S6 shows the calculated form factor ($\mathcal{P} = \frac{\mathcal{F}_m{}^2}{V}$) changes with refractive index of the background material (n_b) for the polysterene opal without (Figure 2a) and with a 3 nm titania ALD coating (Figure 2b). The appearance of the transparency mode (without bandgap) due to the background index change can be used as sensor. For the direct opal, the transparency mode can be induced only if the analyte has the same refractive index as the motif saying $n_b = 1.6$. However, the low concentration analyses in water ($n = 1.33$) or ethanol ($n = 1.36$) solutions etc. can never trigger such transparency mode. But, this can be achieved at $n = 1.4$ for 3 nm (Figure 2b) coating sample. Thus, a little index variation (Δn around 0.04) will change the structure from light reflection to transparency which can be easily tracked by a power meter for the bright or dark mode. This measurement does not require a spectrometer.
